# Supplementary material for: Identification of Novel Substrates for cGMP Dependent Protein Kinase (PKG) through Kinase Activity Profiling to Understand Its Putative Role in Inherited Retinal Degeneration
Source: Int J Mol Sci. 2021 Jan 25;22(3):1180. doi: 10.3390/ijms22031180 (PMC7865299; doi:10.3390/ijms22031180)
Supplement: Supplementary file 1 [file ijms-22-01180-s001.pdf]

Article

Identification of novel substrates for cGMP dependent protein kinase (PKG) through kinase activity profiling to understand its putative role in inherited retinal degeneration

Akanksha Roy [1,2,*], John Groten [1,2], Valeria Marigo [3,4], Tushar Tomar [2] and Riet Hilhorst [2]

[1] Division of Toxicology, Wageningen University and Research, Stippeneng 4, 6708 WE Wageningen, The Netherlands; jgroten@pamgene.com
[2] PamGene International B.V., P.O. Box 1345, 5200 BJ 's-Hertogenbosch, The Netherlands; TTomar@pamgene.com (T.T.); rhilhorst@pamgene.com (R.H.)
[3] Department of Life Sciences, University of Modena and Reggio Emilia, via Campi, 287, 41125 Modena, Italy; vmarigo@unimore.it
[4] Center for Neuroscience and Neurotechnology, University of Modena and Reggio Emilia, via Campi, 287, 41125 Modena, Italy
* Correspondence: akanksha.roy@wur.nl

Supplementary Fig. S1

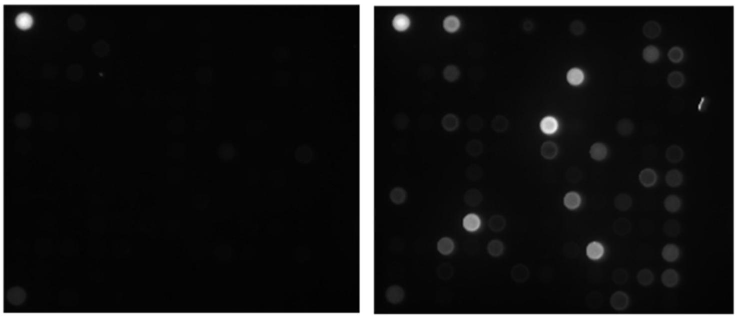

Figure S1. Phosphorylation of peptide microarray by PKGI as function of ATP and cGMP. ATP (0µM, left and 400 µM, right) and cGMP (**0 µM, left and 400 µM, right).**

Supplementary Table S1- Relative signal intensity of peptide substrates as function of enzyme (PKGI or PKGII) concentration

PKGI (ng/array)	F263_454_466	VASP_232_244
0.5	100	100
1	170	217
2.5	275	282
5	297	323

PKGII (ng/array)	F263_454_466	VASP_232_244
5	100	100
10	186	122
20	321	141
30	134	75

Supplementary Fig. S2

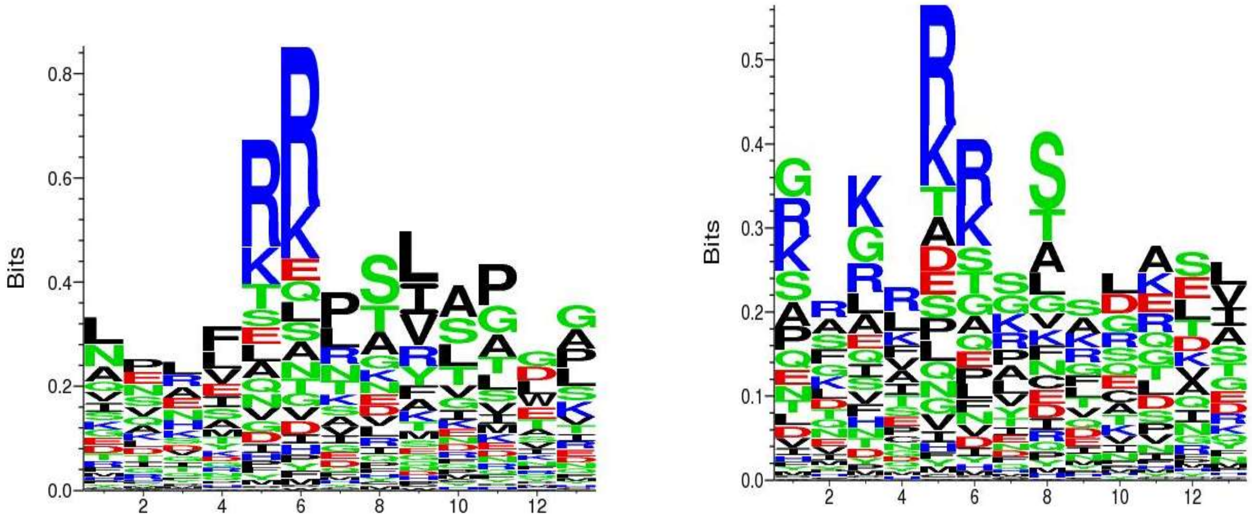

Figure S2. Substrate Logo of PKGI (left) and PKGII (right).

Supplementary Table S2- List of PKA substrates

	PKA Relative Signal Intensity
CFTR_761_773	100
F263_454_466	63
KCNA6_504_516	51
GBRB2_427_439	46
MYPC3_268_280	43
GRIK2_708_720	41
KAP3_107_119	36
TOP2A_1463_1475	29
VTNC_390_402	27
TY3H_65_77	26
STK6_283_295	20
CFTR_730_742	18
NCF1_296_308	17
CDN1A_139_151	16
RS6_228_240	16
SCN7A_898_910	16
CAC1C_1974_1986	15
PTN12_32_44	14
KAP2_92_104	13
KPB1_1011_1023	13
VASP_150_162	12
ADRB2_338_350	10

Supplementary Fig. S3

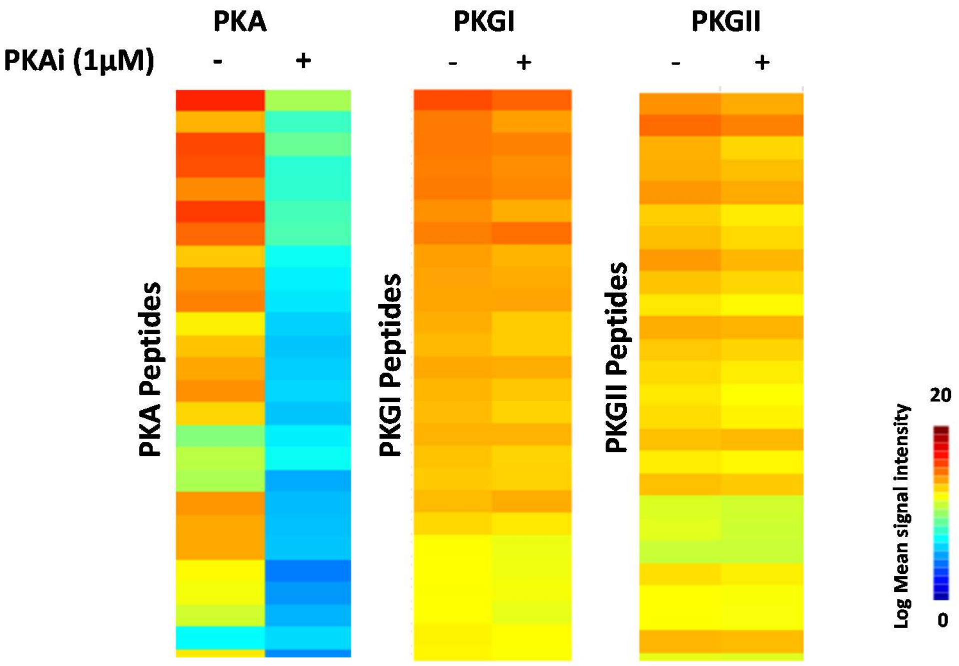

Figure S3. Effect of PKAi on activity of recombinant PKA, PKGI and PKGII.

Supplementary Table S3 PKGI and PKGII substrate scoring

All the experiments were performed on PamChip® 96 plate. The PamChip® 96 contains 96 identical arrays grouped on 24 strips each containing 4 arrays. Every array consists of 142 serine/threonine containing peptides. To minimize the variation between experiments performed on different days and on different PamChip® 96 plates, the experiment setup comprised of a control on each strip with variable concentrations of the modulators on the remaining three arrays of every strip. The effect of modulators was analyzed within each strip and paired with One-Way ANOVA. First, the peptides were selected for ATP dependency. Only the peptides that showed significant increase in phosphorylation with ATP were included in the scoring. The scoring of the peptides for PKGI or PKGII was based on the following parameters with 1 point assigned for every condition met (p value≤ 0.05 for all the conditions, Log fold change> +2 for cGMP or cAMP, > +0.5 for PKG activator and > -0.5 for PKG Inhibitors, and EC_{50} fit R^2> 0.8). For PKGI, the inhibitor Rp-8-Br-PET-cGMPS which is more specific for PKGI was used and for PKGII, the Rp-8-pCPT-cGMPS inhibitor was used.

	p value	Log fold change	EC_{50} fit
cGMP	1	1	1
cAMP	1	1	1
8-Br-cGMP	1	1	
Rp-8-Br-PET-cGMPS	1	1	
Rp-8-pCPT-cGMPS	1	1	
